# Prevalence and Causes of Elective Surgical Cancellations: Findings from a Rural Tertiary Hospital in the Eastern Cape, South Africa

**DOI:** 10.3390/healthcare11020270

**Published:** 2023-01-15

**Authors:** Abongile Sukwana, Busisiwe Mrara, Olanrewaju Oladimeji

**Affiliations:** 1 Anaesthesiology and Critical Care, Faculty of Health Sciences, Walter Sisulu University, Mthatha 5099, South Africa; 2 Department of Public Health, Faculty of Health Sciences, Walter Sisulu University, Mthatha 5099, South Africa

**Keywords:** prevalence, cancellation, elective surgical procedure, rural tertiary hospital

## Abstract

**Background:** Cancellations of elective surgeries adversely affect the patient, hospital staff, facility, and health system. Cancellations potentially result in hospital financial losses, theatre inefficiency, and substandard patient care. A common benchmark for the cancellation rate of elective surgeries is less than five percent, and most operating rooms fall short of this standard. There is a paucity of data on the rates and causes of elective surgical cancellations in rural, resource-limited settings. This study aimed to determine the prevalence of elective surgery cancellations, the causes for such cancellations, and the surgical disciplines most affected at Nelson Mandela Academic Hospital (NMAH). **Methodology:** This was an observational, descriptive, cross-sectional review of operating theatre records from January 2019 to July 2019. The prevalence and main causes of elective case cancellations were determined. The causes were classified, and the most affected surgical departments and patient characteristics were identified. **Results:** The prevalence of elective surgical case cancellations was 14.4% in our hospital, higher than the international benchmark of 5%. Patient-, facility-, and surgical-related factors were the leading causes of cancellations, and avoidable cancellations were mostly surgical- and anaesthetic-related. Ophthalmology was the most affected, followed by gynaecology and general surgery, with plastic surgery being the least affected. The most common patient-related factors were nonattendance and uncontrolled medical conditions, while overbooking was the most common surgical reason. Abnormal investigatory results and unfit status were the most common anaesthetic reasons. Facility-related issues included the lack of theatre time, equipment scarcity or malfunction, and staff unavailability. Most cancellations were unavoidable, but with careful planning, could be avoided. **Conclusion and recommendations:** This study identified challenges with theatre efficiency in a rural, resource-limited setting that call for the cooperation of multidisciplinary teams of surgeons, anaesthetists, nursing staff, and health care policymakers.

## 1. Introduction

Numerous hospitals around the world experience elective surgical case cancellation; this has a negative impact on operating room efficiency, surgical service quality, and patients and their families, and ultimately results in financial losses for the hospital [1,2,3]. Preparation and planning for elective surgical cases necessitate an organised multidisciplinary approach involving the surgical team, theatre staff (including anaesthesiologists), and hospital administration to reduce cancellations and increase theatre efficiency [2,4]. These preparations include patients presenting themselves for admission, a functional operating theatre booking system, patient optimisation, theatre personnel ensuring equipment is available, ward personnel preparing for patients’ admission, and planning for post-operative care [3]. Operating theatres are cost-drivers in hospitals with a substantial investment in specialised equipment and human resources [2,3]. Thus, operating theatre utilisation should be maximised to balance cost and benefit [5]. Elective surgical case cancellation is part of the metrics used to assess theatre efficiency; therefore, it is important to periodically assess theatre efficiency [6]. According to Marcario, this rate should be less than 5% [6].

A surgical case is cancelled when the decision to operate is reversed on the day of the surgery or the day before, or after the patient has been informed of the operation date [7]. The number of cancelled operations and the reasons for them differ depending on the hospital, surgical specialty, and the health system in high-/low-/middle-income countries. Theatre cancellation rates in low-and middle-income countries (LMICs) are typically higher than in high income countries (HIC) [8]. Bhuiyan et al. found a 44.5% cancellation rate in a tertiary hospital in South Africa’s Limpopo Province [1]. Another study found a 26% cancellation rate in a regional hospital in KwaZulu Natal [9]. High cancellation rates have also been reported in other African countries, including 37% in Burkina Faso [2] and 28.8% in a Ugandan tertiary hospital [10]. There is a dearth of information regarding the elective case cancellation rates at the rural Nelson Mandela Academic Hospital (NMAH). Rural hospitals face difficulties with patient transportation logistics and pre-operative preparation opportunities. In addition to limited resources, these hospitals serve a large rural patient population. The purpose of this study was to determine the cancellation rates, the reasons for elective surgical case cancellations, and the surgical specialties most affected at NMAH. High cancellation rates for elective surgical procedures reduce operating room efficiency and negatively impact patients, staff, hospitals, and the health care system. This study was conducted as part of an initiative to evaluate operating room (OR) efficiency and the effect of elective case cancellations on patient care quality.

## 2. Materials and Methods

### 2.1. Study Design

This was an observational, descriptive, cross-sectional study in which a retrospective review of theatre records from January 2019 to July 2019 was performed. For this study, a cancellation was defined as a case that appears on the OT booking book generated and booked before 14H00 on the previous day but subsequently not carried out on the particular day due to whatever reasons stated. 

### 2.2. Study Setting 

This study was conducted in the NMAH main operating theatre complex with six operating rooms. One theatre is mainly delegated for emergencies and operates for 24 h a day and seven days a week. The remaining five operate on weekdays from 07H30 to 16H00. General surgery, gynaecology, neurosurgery, plastic surgery, maxillofacial surgery, ophthalmology, ear, nose, and throat surgery, paediatric surgery, urology, and cardiothoracic surgery use these theatres for elective work, according to a weekly allocation. The theatre policy stipulates that anaesthetists have to assess all elective cases on the day before the procedure. 

NMAH is a government-funded public hospital situated in Mthatha, Eastern Cape Province, South Africa, and is the only tertiary hospital in the northeastern part of the Eastern Cape. The hospital is situated in the rural portion of the province and serves a catchment area of roughly 3 million people with 736 beds. Most of the population consists of rural, African, impoverished patients of low socioeconomic status. NMAH is the only facility in the OR Tambo District that offers tertiary surgical, medical, allied health, and trauma services.

### 2.3. Patient Selection 

Inclusion criteria:

- Elective surgical cases that were cancelled at NMAH main theatre from January 2019 to July 2019.

Exclusion criteria:

- Obstetric cases (cases carried out at labour ward theatre);
- Orthopaedic cases (cases carried out at an off-site orthopaedic unit);
- All emergency cases (cases carried out in a designated theatre).

### 2.4. Sampling Method and Data Source

We conducted a retrospective, sequential review of all cancelled elective surgery cases, as recorded in the OT booking book. Patients who met the study’s inclusion criteria during the specified period were included. A proportional sampling strategy was implemented per surgical unit to ensure that the data volume from each department was adequately represented.

The records included the date, the surgical department, the patient’s demographics (name, file number, diagnosis, age, and gender), the procedure to be performed, the patient’s time of arrival in the reception area, the time the patient entered the operating room, and whether the procedure was completed or cancelled, as well as the reason for cancellation.

### 2.5. Data Collection and Variables of Interest

A data collection form was used to extract unaltered data from theatre records filled by nurses working in the reception area of the theatre. 

The information collected was age, gender, diagnosis, the procedure to be performed, department and status, i.e., whether it was performed or cancelled, and the reason for cancellation, including the category. Each patient was assigned a serial number for de-identification and the patients’ names were not included in the data for confidentiality purposes. The reasons for cancellation were categorised into patient-, surgical-, anaesthetic-, and facility-related factors and further stratified into avoidable or unavoidable.

### 2.6. Data Analysis

Data were assessed for completeness and accuracy and entered into Microsoft Excel on a password-protected personal computer only accessible to the researcher. Once cleaned, they were imported for analysis using Statistical Package for Social Sciences (SPSS) software.

The continuous variables were presented as means and standard deviations, and Student’s *t*-test was used for their comparison. For the categorical variables, proportions (percentages) of interest were assessed, and a chi-square test was used to test for comparison. A *p*-value < 0.05 is regarded as statistically significant.

A prevalence rate of > 5 percent was regarded as high for elective surgical case cancellation. The prevalence rate for the study period was calculated using the formula below.

$$\mathrm{Prevalence}=\frac{Total\;number\;of\;cancelled\;electives}{Total\;booked\;elective\;cases}\times 100$$

## 3. Results 

### 3.1. Demographics of the Study Participants

A total of 2962 elective surgical cases were booked, with 428 elective theatre cases being cancelled at Nelson Mandela Academic hospital from January 2019 to July 2019.

The highest percentage of patients (38.59%, n= 164) were observed in the age range 36–64 years, 28.9% were >65 years, followed by those aged 19–35 years, comprising 19.76%, as demonstrated in Table 1.

The mean age of cases was 47.72 ± 23.94 years. The study observed more female participants than males (55.37% vs. 44.63%) with a female:male ratio of 1.24:1.

### 3.2. Prevalence Rate of Cancellation of Elective Surgical Cases 

**Figure 1:**
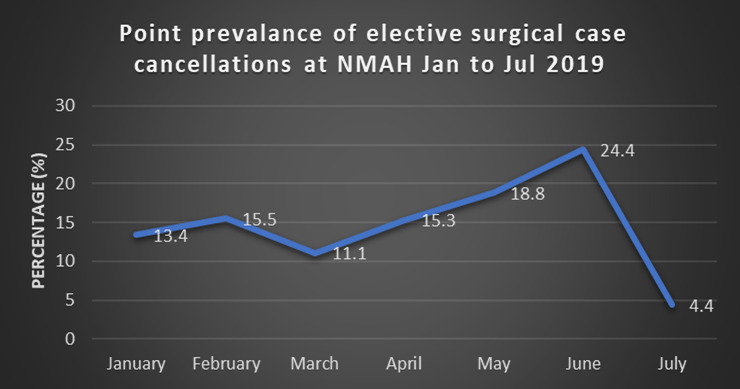
Comparison of the prevalence of cancellations by month.

The overall prevalence rate of elective surgical case cancellation in NMAH from January 2019 to July 2019 was 14.4%. As shown in Figure 1, the prevalence rate of cancellation of elective surgical cases progressively increased from January (13.4%) to June, with the highest rate at 24.4% in June, before a considerable decline in July, with a prevalence rate of 4.4%. Table 2 illustrates the number of booked elective cases per month, and the mean average of elective surgical cases booked per month was 423. The month of July showed the highest number of cases booked (574), with fewer cases cancelled (4.4%). 

### 3.3. Causes of Cancellation of the Study Participants

This was presented in four categories: patient-related, anaesthetic-related, surgical-related, and facility-related factors. 

#### 3.3.1. Patient-Related Factors

The most presented reason under the *patient-related* category was a failure to show up, with 73.65% (123). Others were hypertension, with 10.18% (17), patient eating, accounting for 6.59% (11), upper/lower respiratory tract infection, with 3.59% (6), and patient refusal, accounting for 2.40% (4), as shown in Table 3.

#### 3.3.2. Surgical-Related Factors 

The most presented reason under the *surgical-related* factors was overbooking, at 34.21% (39). Others were malfunction or lack of equipment, accounting for 18.42% (21), postponed, with 14.91% (17), change in diagnosis, with 14.03% (16), poor workup, accounting for 8.7% (10), and cases cancelled by the surgeon, accounting for 5.26% (6), as shown in Table 4.

#### 3.3.3. Anaesthesia-Related Factors 

**Figure 2:**
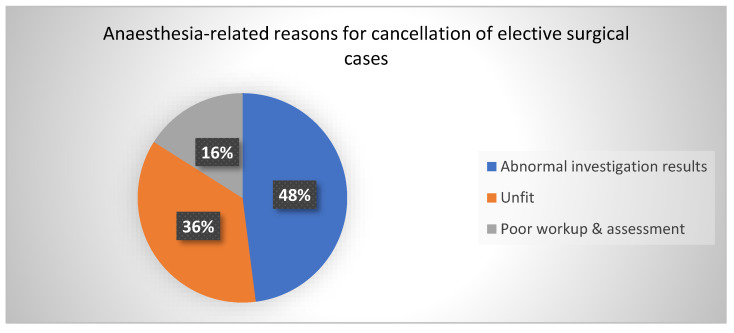
Anaesthesia-related factors.

The most presented reason under the *Anaesthesia-related* category was abnormal investigation results, with 50.0% (12). Others were the patient being unfit for anaesthesia, with 37.5% (9), and poor workup and assessment, accounting for 16.67% (4), as shown in Figure 2.

#### 3.3.4. Facility-Related Factors

The most presented reason under facility-related was the time factor (theatre time running out), accounting for 46.34% (57). Others were the lack of steam for sterilising instruments, with 20.33% (25), unavailability of nursing staff, accounting for 2.20% (15), having no linen, with 4.88% (6), and unavailability of pre- or post-op beds, with 4.88% (6), as shown in Table 5.

### 3.4. Avoidable and Unavoidable Reasons for Cancellation

Table 6 demonstrates that most elective surgical cases (59.35%, n-254) were cancelled due to unavoidable reasons based on pre-existing definitions. 

Avoidable causes were defined as cancellations or delays that occurred because of situations that existed before the day of surgery and could have been avoided with careful review and communication between patients and staff.

Unavoidable causes were defined as delays or cancellations that could not have been avoided even with adequate review or communication between patients and staff.

### 3.5. Most Affected Surgical Departments, Surgical Procedures, and Frequently Cancelled Patients

#### 3.5.1. Surgical Departments 

**Figure 3:**
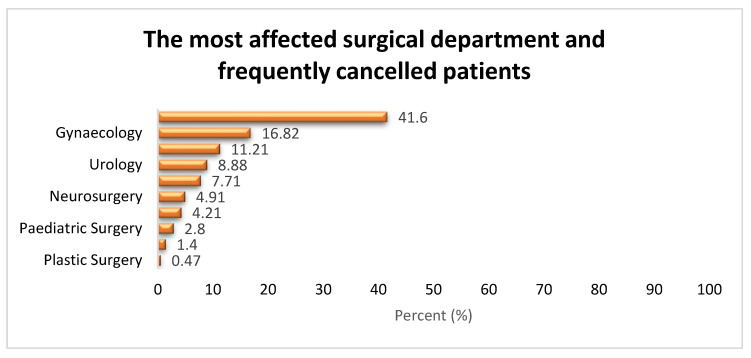
Most affected surgical departments.

The most affected surgical department was ophthalmology, with 41.59% (n-178). Other notably affected departments included gynaecology, with 16.82% (72), general surgery, accounting for 11.21% (48), urology, with 8.88% (38), and ENT, with 7.71% (33), as shown in Figure 3.

#### 3.5.2. Surgical Procedures 

As shown in Table 7, the most affected surgical procedure was Avastin injection, accounting for 17.99% (77), followed by excisional biopsy, with 10.51% (n-45), SICS and IOL, with 8.41% (n-36), total abdominal hysterectomy, comprising 4.21% (n-18), thoracotomy, with 3.74% (n-16), DD&C, with 3.50% (n-15), and optical urethrotomy, accounting for 2.57% (n-11). 

#### 3.5.3. Sociodemographic Features of Frequently Cancelled Patients

As shown in Table 8, no statistically significant association was observed between socio-demographic characteristics and any categories of elective surgical cancellations (*p* > 0.05). 

### 3.6. Surgical Factors Per Category of Elective Surgical Cancellations

#### 3.6.1. Surgical Department

Avoidable surgical cancellations that were statistically significant were observed most in maxillofacial surgery (66.67%), urology (63.16%), and gynaecology (62.50%) (*p* = 0.001), as shown in Table 9.

#### 3.6.2. Surgical Procedures

Avoidable surgical cancellations as stratified according to the surgical procedures were observed mostly for laparotomy and dye test (100.0%), D&C (80.0%), and optical urethrotomy (72.73%) (*p* = 0.001), and this was statistically significant, as demonstrated in Table 10.

### 3.7. Causes of Cancellation Per Category of Elective Surgical Cancellations

The statistically significant causes of avoidable surgical cancellations were observed mostly in the surgically related (99.12%) and anaesthesia-related (66.67%) causes (*p* = 0.001), as shown in Table 11. 

## 4. Discussion

In this study 428 elective surgery were cancelled out of the 2962 booked elective cases during the study period; most cancellations were female, constituting 55% of the total cases; and the majority of cancelled patients were between 36 and 64 years. 

### 4.1. Prevalence of Elective Surgical Case Cancellation

The prevalence of elective surgical case cancellation was found to be 14.4% in the six month study period. This is higher than the internationally quoted benchmark of less than 5% [6], and the rate is similar to two other studies in South Africa that showed cancellation rates ranging from 26% to 44,5% [1,9]. It is challenging to have a clear picture of the prevalence rate of elective surgical case (ESC) cancellations in most parts of South Africa due to the paucity of studies conducted. The prevalence rate of ESC cancellation in our study is similar to the findings of a meta-analysis of studies of cancellations that reported a global prevalence of 18%, with the highest cancellations being in sub-Saharan Africa, at 36% [11]. 

This rate is also similar to other African studies where the prevalence ranged between 21% and 37% [2,8,10,12]. This is in line with the high cancellation rates reported in other LMICs [8]. NMAH, as a rural tertiary hospital, serves a large catchment area, with most of its referring hospitals being located at far distances compared with other tertiary hospitals in the country. This can result in transportation issues due to poor road conditions and poor communication between patients and healthcare providers. There is also an increased number of emergency cases due to poor access to elective surgery, which results in disease progression and complicated presentations. 

The cancellation rates were found to vary by month. June showed the highest percentage of cancellations, at 24.4%, while July, had the lowest, at 4.4%, despite having a higher number of bookings than the other months. An explanation for the discrepancy is the period of school holidays when more children show up for operations. Another factor was the unavailability of steam to sterilise theatre instruments in June, which could be related to procurement processes, budgeting, and health system planning issues. These reasons were difficult to determine in this retrospective review and would need to be explored further. 

A lower rate of ESC cancellation is reported in HICs compared to LMICs, namely 0.37% vs 22% [13,14,15]. The possible reasons for the discrepancy include socioeconomic conditions, adequate infrastructure, staffing and equipment in HICs. Also, HICs have effective communication systems between the hospital and patients, ample transport for patients to reach the hospital, and less patient load, as there is a better spread of healthcare services [14,16].

### 4.2. Causes of Elective Surgical Case Cancellation

The top three causes of cancellations were patient-related, facility-related, and surgical-related factors, and there was an overlap between these categories. Surgical-related factors accounted for the majority of avoidable causes (99.12%), followed by anaesthetic-related factors (66.67%), and these findings were statistically significant. The leading causes of avoidable surgical- and anaesthetic-related cancellations were overbooking, equipment malfunction/unavailability, abnormal investigation results, and unfit patients for anaesthesia. The reason for overbooking is that the combined average time of booked cases exceeded the allocated eight hours for electives per weekday. Insufficient operative time in the facility was also a factor, contributing to half of the facility-related cancellations, with unanticipated theatre delays being the most common reason. These facility-related causes were unavoidable; however, through careful evaluation and planning, they can be avoided. These results are comparable to those of a study conducted in Durban, South Africa, where 41% of cancellations were due to insufficient operative time [9]. A Hong Kong audit conducted by Chiu revealed that a lack of operational time is the leading cause of facility-related cancellations [13]. 

Logistics related to admission and pre-operative preparation are difficult in rural hospitals. Patients are admitted the day before surgery, and blood test results may not be available until the morning of surgery, leading to the late discovery of unfit status. Some patients’ medical conditions deteriorate overnight, rendering them unfit for anaesthesia on the morning of surgery.

We found that almost three-quarters of patients did not show up for their surgical procedures, and the majority of these cancellations were unavoidable. This was also the case in three studies in the Middle East, Finland, and Nigeria, where patient-related factors contributed 67%, 54.7%, and 60.8%, respectively [15,17,18]. In northwestern Nigeria, Gajida and colleagues found that the main patient-related cause was patients not showing up for procedures, with the root cause being unknown [18]. In our study, the reasons for patients not showing up were unknown but could be attributed to poor communication, socioeconomic status, and lack of transport. 

In this study, cancellations within the control of the surgical department, such as overbooking, the failure or lack of equipment, postponement, and alteration in the diagnosis, were largely avoidable.

Reasons for cancellation of elective surgical procedures vary from hospital to hospital [14,19]. Numerous complex factors and dynamics influence hospital cancellation rates, making hospital comparisons difficult [20]. In a study carried out in a hospital in Limpopo Province, South Africa, the most common reasons for cancellations were the lack of a dedicated emergency operating theatre, equipment, and consumables [1]. Due to the availability of a dedicated emergency theatre, the above factors did not significantly contribute to cancellations in our study. However, the malfunctioning or lack of equipment, lack of personnel, lack of steam for the sterilisation of instruments, postponement or cancellation of cases by surgeons, suboptimal medical conditions (such as hypertension), and lack of post-operative beds did significantly contribute to cancellations. These reasons illustrate the suboptimal organisation of perioperative patient management. The lack of operating facilities or equipment, lack of skilled surgeons, and alterations in patient medical conditions are cited as common causes on a global scale [11]. In addition, several studies have shown that poor communication between patient and physician, as well as between patient and hospital, and communication within the hospital significantly contribute to cancellations despite booking [5,15,17].

### 4.3. Avoidable vs. Unavoidable Category of Elective Surgical Cancellation

Our definitions of avoidable vs. unavoidable cancellations were based on the opportunity to intervene to prevent cancellation, mainly on those situations that existed before the day of surgery and could have been avoided by careful planning and communication. Admittedly, this classification is not always clear, as communication with patients is not easy in resource-limited, rural settings. Patient no-shows are classified as an unavoidable cancellation in these settings, whereas they could be avoided with telecommunication in an adequately resourced setting. 

We found that 59.4% of the cancellations were unavoidable, while 40.7% were avoidable. This is comparable to a Nigerian study in which 60.8% of cases were cancelled due to no-shows and classified as unavoidable [18]. Typical unavoidable causes in our study were patients not showing up, patients eating prior to OT, and operating time running out, all of which are preventable with improved planning. Other researchers have found more avoidable than unavoidable cancellations. Lankoande’s study in Burkina Faso classified 89.5% of cancellations as avoidable, with patient nonattendance being the leading cause, notably classifying nonattendance as avoidable [2]. This illustrates the lack of standardisation and variation in the definitions of avoidable and unavoidable cancellations across studies. Several studies classify most cancellations as avoidable [2,8,11,12].

Most cancellations in our study were due to nonclinical causes, making them potentially avoidable with careful planning. A multidisciplinary approach to elective surgical case preparation and planning from the patient, surgical team, theatre staff (including anaesthetists), and hospital administration is likely to reduce theatre cancellations [2,4]. Using electronic patient platforms to cancel or reschedule appointments could be beneficial in minimising the wastage of booked theatre time.

### 4.4. Surgical Disciplines and Procedures

Most elective surgery cancellations occurred in ophthalmology, gynaecology, general surgery, urology, and ENT. The high cancellation rate in ophthalmology is likely due to the department’s large patient volume, with most patients arriving on the morning of the scheduled procedure. Due to poor communication, a number of patients do not appear for their scheduled surgical procedures. This suggests the need to improve the booking system and patient communication. Other studies found general surgery, gynaecology, and orthopaedics to be the most frequently affected specialties [2,12,18]. In a study conducted at a tertiary care rural hospital in India, Naik and colleagues discovered that ophthalmology had the fewest elective surgical case cancellations due to a dedicated and efficient operating theatre [5]. These contradictory findings demonstrate the complexity of ESC and how it varies from hospital to hospital. 

In our tertiary hospital, small procedures were the most likely to be cancelled, such as injections, excisional biopsies, anal examination under anaesthesia, the cauterisation of genital warts, and cone biopsies. These are short procedures that could be performed under local anaesthesia or in minor operating rooms to free up the main operating room for more complex cases. Again, this calls for a need for reorganisation and decentralisation of minor surgical services.

## 5. Strengths and limitations 

### 5.1. Strength

This was the first study of its kind in the hospital, and we compared and assessed the burden of elective surgical case cancellation using international benchmarks. The study sheds light on how patients and health providers contribute to the cancellation of elective surgical procedures in our centre.

### 5.2. Limitations

This was a single-centre study illustrating the challenges specific to our hospital and not necessarily generalisable to other settings. In our context, we applied pre-existing definitions of avoidable and unavoidable causes of cancellations. This could have led to most avoidable causes being classified as unavoidable.

In this retrospective audit, poor record keeping, and the possibility of missing values were a challenge and could be minimised with sufficient sample size. Further, we could not interview patients to investigate the causes of nonappearance for surgical procedures. Follow-up information to determine whether patients were rescheduled was unavailable.

## 6. Conclusions

The rate of elective surgical case cancellation at Nelson Mandela Academic hospital is fourteen percent, higher than the international benchmark of less than five percent, confirming that the rate of elective surgical case cancellations is higher in LMIC health systems. There was a monthly variation in prevalence rates due to the differing volumes of booked cases. The ophthalmology department was the most affected, followed by gynaecology and general surgery, with plastic surgery being the least affected. Minor procedures that can be performed in a minor theatre were mostly cancelled. The leading categories of cancellation were patient-, facility-, and surgical-related factors. Failure of patients to show up, uncontrolled medical conditions, overbooking, and OT time running out were the leading causes. In our study, the majority of cancellations were unavoidable based on existing definitions, but with careful assessment and planning, these are avoidable. Our study proves the anticipated status quo and has similarities to other local studies and developing countries. 

## 7. Recommendations

The reorganisation and decentralisation of surgical services for minor procedures could reduce overbooking and improve the availability of theatre time for major cases.

Improved communication and planning between patients and perioperative teams could improve scheduling. This includes a dynamic theatre booking system with data capture, which enables optimising pre-operative procedures, planning equipment needs, and estimating the duration of each procedure based on existing data. In addition, having a digital patient booking system that allows patients to timeously confirm or cancel their appointments will enable the fruitful use of theatre time.

Elective surgical case cancellations should be regarded as adverse events with regular auditing and determination of causes and solutions. Future research should assess the implementation and compliance of the proposed recommendations. This would help to assess our overall theatre efficiency and improvement in the ESC cancellation rate.

## Figures and Tables

**Table 1:** Selected sociodemographic characteristics of the study participants (n = 428).

Variables	Frequency	Percent
Age (years) (n = 425) ^γ^		
0–1	14	3.29
2–12	24	5.65
13–18	16	3.76
19–35	84	19.76
36–64	164	38.59
>65	123	28.94
*Mean (SD)*	47.72 ± 23.24 years	
Gender (n = 137) ^γ^		
Female	237	55.37
Male	191	44.63
*Ratio*	1.24: 1	
^γ^ = Missing *Values applicable*		

**Table 2:** Number of elective surgical cases booked at NMAH per month.

Variables	Frequency
Months	*n* = 2962
January	216
February	386
March	449
April	404
May	453
June	480
July	574

**Table 3:** Patient-related factors.

Variables	Frequency	Percent
Patient-related (n = 167) ^γ^		
Failure to show up	123	73.65
Uncontrolled hypertension	17	10.18
Patient eating	11	6.59
Upper or lower respiratory tract infection	6	3.59
Patient refusal	4	2.40
Uncontrolled diabetes mellitus	2	1.20
Atrial fibrillation	1	0.60
Collapsed	1	0.60
Deteriorated condition	1	0.60
Asthma	1	0.60
^γ^ = Missing *Values applicable*		

**Table 4:** Surgical-related factors.

**Variables**	**Frequency**	**Percentage**
Surgical-related (n = 114) ^γ^		
Overbooking	39	33.21
Malfunction or lack of equipment	21	18.42
Postponed	17	14.91
Change of diagnosis	16	14.03
Poor workup	10	8.70
Cancelled by surgeon	6	5.26
Surgical staff unavailable	3	2.63
Does not belong to the firm	1	0.87
Late start	1	0.87
^γ^ = Missing *Values applicable*		

**Table 5:** Facility-related factors.

Variables	Frequency	Percent
Facility-related (n = 123) ^γ^		
Time factor	57	46.34
No steam	25	20.33
No nursing staff	15	12.20
No Linen	6	4.88
Unavailability of pre- or post-op beds	6	4.88
Prioritisation of other cases including emergencies	4	3.25
Unavailable or malfunctioning equipment	3	2.44
Delay due to death on the table	3	2.44
No water	1	0.81
	1	0.81
Air-conditioning malfunction	1	0.81
Given clexane (enoxaparin) in ward	1	0.81
^γ^ = Missing *Values applicable*		

**Table 6:** Category of cancellation of the study participants (n = 428).

Variables	Frequency	Percentage
Category of cancellation		
Avoidable	174	40.65
Unavoidable	254	59.35

**Table 7:** Most affected surgical procedures (n = 428).

Variables	Frequency (n)	Percentage (%)
Surgical Procedures		
Avastin injection	77	17.99
Excisional biopsy	45	10.51
Small incision cataract surgery and intraocular lens (SICS & IOL)	36	8.41
Total abdominal hysterectomy	18	4.21
Thoracotomy	16	3.74
Dilatation and curettage (D&C)	15	3.50
Optical urethrotomy	11	2.57
Cauterisation of corneal vessels	9	2.10
Cone biopsy	9	2.10
Examination under anaesthesia (EUA) of anus	9	2.10
Transurethral resection prostate (TURP)	9	2.10
Orchidopexy	8	1.87
Herniotomy	5	1.17
Laparotomy and dye test	5	1.17
Others	156	36.44

**Table 8:** Sociodemographic features associated with categories of reasons for cancellation (n = 428).

Variables	Category of Elective Surgical Cancellations (Freq %)		Total	Chi-Square, *p*-Value
	Avoidablen n = 174	Unavoidablen n = 254		
**Age**				χ2 = 4.53, *p* = 0.476
0–1 yrs	8 (57.14)	6 (42.86)	14 (100.0)	
2–12 yrs	7 (29.17)	17 (70.83)	24 (100.0)	
13–18 yrs	7 (43.75)	9 (56.25)	16 (100.0)	
19–35 yrs	38 (45.24)	46 (54.76)	84 (100.0)	
36–64 yrs	61 (37.20)	103 (62.80)	164 (100.0)	
>65 yrs	51 (41.46)	72 (58.54)	123 (100.0)	
**Gender**				χ2 = 0.00, *p* = 0.976
Male	78 (40.84)	113 (59.16)	191 (100.0)	
Female	96 (40.51)	141 (59.49)	237 (100.0)	

**Table 9:** Categories of cancellations per surgical department.

Variables	Category of Elective Surgical Cancellations (Freq %)		Total	Fisher’s Exact *p*
	Avoidablen n = 174	Unavoidablen n = 254		
Surgical Department				*p* = 0.001 ^µ^*
Ophthalmology	41 (23.03)	137 (76.97)	178 (100.0)	
Gynaecology	45 (62.50)	27 (37.50)	72 (100.0)	
General Surgery	27 (56.26)	21 (43.75)	48 (100.0)	
Urology	24 (63.16)	14 (36.84)	38 (100.0)	
Ear, nose, and throat surgery (ENT)	13 (39.39)	20 (60.61)	33 (100.0)	
Neurosurgery	9 (42.86)	12 (57.14)	21 (100.0)	
Cardiothoracic surgery	6 (33.33)	12 (66.67)	18 (100.0)	
Paediatric surgery	4 (33.33)	8 (66.67)	12 (100.0)	
Maxillofacial surgery	4 (66.67)	2 (33.33)	6 (100.0)	
Plastic surgery	1 (50.0)	1 (50.0)	2 (100.0)	

* Statistically significant (*p* < 0.05); µ = Fisher’s exact *p* (recommended for values < 5).

**Table 10:** Surgical procedures per category of elective surgical cancellations (n = 428).

Variables	Category of Elective Surgical Cancellations (Freq %)		Total	Fisher’s Exact *p*
	Avoidablen n = 174	Unavoidablen n = 254		
Surgical Procedure				*p* = 0.001 ^µ^*
Avastin injection	10 (12.99)	67 (87.01)	77 (100.0)	
Excisional biopsy	8 (17.78)	37 (82.22)	45 (100.0)	
SICS and IOL	19 (52.78)	17 (47.22)	36 (100.0)	
Total abdominal hysterectomy	10 (55.56)	8 (44.44)	18 (100.0)	
Thoracotomy	5 (31.25)	11 (68.75)	16 (100.0)	
DD&C	12 (80.0)	3 (20.0)	15 (100.0)	
Optical urethrotomy	8 (72.73)	3 (27.27)	11 (100.0)	
Cauterisation of corneal vessels	2 (22.22)	7 (77.78)	9 (100.0)	
Cone biopsy	3 (33.33)	6 (66.67)	9 (100.0)	
EUA anus	6 (66.67)	3 (33.33)	9 (100.0)	
TURP	6 (66.67)	3 (33.33)	9 (100.0)	
Orchidopexy	4 (50.0)	4 (50.0)	8 (100.0)	
Herniotomy	2 (40.0)	3 (60.0)	5 (100.0)	
Laparotomy and dye	5 (100.0)	0 (0.0)	5 (100.0)	

* Statistically significant (*p* < 0.05); µ = Fisher’s exact *p* (recommended for values < 5).

**Table 11:** Causes of cancellation per category of elective surgical cancellations (n = 428).

Variables	Category of Elective Surgical Cancellations (Freq %)		Total	Fisher’s Exact *p*
	Avoidablen n = 174	Unavoidablen n = 254		
Causes of cancellation				*p* = 0.001 ^µ^*
Patient-related	16 (9.58)	151 (90.42)	167 (100.0)	
Surgical-related	113 (99.12)	1 (0.88)	114 (100.0)	
Anaesthesia-related	16 (66.67)	8 (33.3)	24 (100.0)	
Facility-related	29 (23.57)	94 (76.42)	123 (100.0)	

* Statistically significant (*p* < 0.05), µ = Fisher’s exact *p* (recommended for values < 5).

## Data Availability

The clean datasets used for this study are not publicly available. However, these could be shared by the authors upon reasonable request and with permission of the Nelson Mandela Academic Hospital and Walter Sisulu University.

## References

[1] Bhuiyan M.M.Z.U., Mavhungu R., Machowski A. (2017). Provision of an emergency theatre in tertiary hospitals is cost-effective: Audit and cost of cancelled planned elective general surgical operations at Pietersburg Hospital, Limpopo Province, South Africa. S. Afr. Med, J..

[2] Lankoandé M., Bonkoungou P.K.K., Kaboré A.F.R., Ouangré E., Savadogo Y., Bougouma C.T., Sanou J., Ouedraogo N., Pendeville P. (2017). Economic and psychological burden of scheduled surgery cancellation in a sub-Saharan country (Burkina Faso). S. Afr. J. Anaesth. Analgesia..

[3] Kumar R., Gandhi R. (2012). Reasons for cancellation of operation on the day of intended surgery in a multidisciplinary 500 bedded hospital. J. Anaesthesiol. Clin. Pharmacol..

[4] Nanjappa N., Kirti K., Kabeer K., Smile S. (2014). Elective Surgical Case Cancellation–An Audit. Int. J. Curr. Res. Review..

[5] Naik S.V., Dhulkhed V.K., Shinde R.H. (2018). A prospective study on operation theater utilization time and most common causes of delays and cancellations of scheduled surgeries in a 1000-bedded tertiary care rural hospital with a view to optimize the utilization of operation theater. Anesth. Essays Res..

[6] Macario A. (2006). Are your hospital operating rooms "efficient"? A scoring system with eight performance indicators. Anesthesiology.

[7] Ezike H., Ajuzieogu V., Amucheazi A. (2011). Reasons for Elective Surgery Cancellation in a Referral Hospital. Ann. Med. Health Sci. Res..

[8] Desta M., Manaye A., Tefera A., Worku A., Wale A., Mebrat A., Gobena N. (2018). Incidence and causes of cancellations of elective operation on the intended day of surgery at a tertiary referral academic medical center in Ethiopia. Patient Saf. Surg..

[9] Asmal I.I., Keerath K., Cronje L. (2019). An audit of operating theatre utilisation and day-of-surgery cancellations at a regional hospital in the Durban metropole. S. Afr. Med. J..

[10] Ogwal A., Oyania F., Nkonge E., Makumbi T., Galukande M. (2020). Prevalence and Predictors of Cancellation of Elective Surgical Procedures at a Tertiary Hospital in Uganda: A Cross-Sectional Study. Surg. Res. Pract..

[11] Abate S.M., Chekole Y.A., Minaye S.Y., Basu B. (2020). Global prevalence and reasons for case cancellation on the intended day of surgery: A systematic review and meta-analysis. Int. J. Surg. Open.

[12] Chalya P.L., Gilyoma J.M., Mabula J.B., Simbila S., Ngayomela I.H., Chandika A.B., Mahalu W. (2011). Incidence, causes and pattern of cancellation of elective surgical operations in a university teaching hospital in the Lake Zone, Tanzania. Afr. Health Sci..

[13] Chiu C.H., Lee A., Chui P.T. (2012). Cancellation of elective operations on the day of intended surgery in a Hong Kong hospital: Point prevalence and reasons. Hong Kong Med. J..

[14] Dhafar K.O., Ulmalki M.A., Felemban M.A., Mahfouz M.E., Baljoon M.J., Gazzaz Z.J., Baig M., Hamish N.M., AlThobaiti S.A., Al-Hothali F.T. (2015). Cancellation of operations in Saudi Arabian hospitals: Frequency, reasons and suggestions for improvements. Pak. J. Med. Sci..

[15] Morris A.J., McAvoy J., Dweik D., Ferrigno M., Macario A., Haisjackl M. (2017). Cancellation of Elective Cases in a Recently Opened, Tertiary/Quaternary-Level Hospital in the Middle East. Obstet. Anesth. Dig..

[16] Hovlid E., Bukve O., Haug K., Aslaksen A.B., von Plessen C. (2012). A new pathway for elective surgery to reduce cancellation rates. BMC Health Serv. Res..

[17] Hänninen-Khoda L., Koljonen V., Ylä-Kotola T. (2018). Patient-related reasons for late surgery cancellations in a plastic and reconstructive surgery department. JPRAS Open.

[18] Takai I.U., Gajida A.U., Nuhu Y.N. (2016). Cancellations of elective surgical procedures performed at a Teaching Hospital in North-West Nigeria. J. Med. Trop..

[19] Kaddoum R., Fadlallah R., Hitti E., El-Jardali F., Eid G.E. (2016). Causes of cancellations on the day of surgery at a Tertiary Teaching Hospital. BMC Health Serv. Res..

[20] van As A.B., Brey Z., Numanoglu A. Improving Operating Theatre Efficiency in South Africa 2011. http://www.scielo.org.za/scielo.php?script=sci_arttext&pid=S0256-95742011000700008.

